# Functionalized Magnetic Carbon Nanoparticles Efficiently Break Water-in-Heavy Oil Emulsions

**DOI:** 10.3390/ma19122584

**Published:** 2026-06-15

**Authors:** Jinlong Gao, Lulu Yan, Jun Ma

**Affiliations:** 1 College of Energy and Chemical Engineering, Shaanxi Polytechnic University, Xianyang 712000, China; 2 Department of Chemical Engineering, School of Chemistry and Chemical Engineering, Guizhou University, Guiyang 550025, China

**Keywords:** magnetic carbon nanoparticles, water-in-heavy oil emulsions, demulsification, mechanism

## Abstract

Achieving efficient demulsification of water-in-heavy oil (W/HO) emulsions remains a critical issue that urgently needs to be addressed in the heavy oil industry. Despite being a new generation of green demulsification materials, magnetic carbon nanoparticles still suffer from low demulsification efficiency when applied to water-in-heavy oil emulsions. Herein, polyethyleneimine-modified magnetic carbon nanoparticles (P-MCNs) were successfully prepared via a surface functionalization strategy. The demulsification performance of P-MCN in water-in-heavy oil (W/HO) emulsions was evaluated via the standard bottle test. The results demonstrated that P-MCN (500 ppm) achieved effective water removal within 60 min at 50 °C. Microscopic visualization characterization revealed that the efficient water removal from W/HO emulsions by P-MCN is attributed to its high interfacial activity. Specifically, P-MCN can rapidly migrate to the heavy oil–water interface and effectively disrupt the interfacial film through electrostatic interactions and hydrogen bonding, thereby achieving efficient demulsification of W/HO emulsions. This study provides a solid theoretical foundation for the further development of magnetic carbon nanoparticles with higher demulsification efficiency for applications in the petroleum industry.

## 1. Introduction

Heavy oil, as an important component of petroleum resources, plays a crucial strategic role in alleviating the increasing shortage of conventional light crude oil through its efficient extraction and processing [1,2]. However, water is inevitably introduced during the extraction, transportation, and refining processes of heavy oil, resulting in the formation of highly stable oil–water emulsions [3,4,5]. These emulsions, characterized by rigid interfacial films and high viscosity [6], hinder oil–water separation, thereby exacerbating equipment corrosion, increasing energy consumption and processing costs [7], and severely impairing crude oil quality and refining efficiency [8]. Therefore, achieving efficient and rapid demulsification of heavy oil emulsions to recover valuable oil resources and purify wastewater remains a critical technical challenge in the petroleum industry.

At present, demulsification technologies developed in both industry and academia mainly include biological, physical, and chemical methods [9,10]. Biological methods are environmentally friendly but suffer from slow action, while physical methods (such as centrifugation, heating, and electric field treatment) often involve high energy consumption and complex equipment [11,12]. Compared with these methods, chemical demulsification has become the most widely applied strategy due to its operational simplicity, broad applicability, and high efficiency [13,14]. The essence of this approach lies in the design and utilization of demulsifiers, which mainly include surfactants, polymers, and nanomaterials. In recent years, magnetic Fe_3_O_4_ nanoparticles have attracted considerable attention as green demulsification materials owing to their unique superparamagnetism, high specific surface area, and the ability to be rapidly recovered and reused under an external magnetic field [15,16,17]. However, pristine Fe_3_O_4_ nanoparticles suffer from high surface energy and a strong tendency to aggregate, and their poor interfacial compatibility with heavy oil emulsions leads to limited demulsification efficiency, which hinders their practical application [18]. The fundamental limitation lies in their insufficient interfacial activity. Therefore, precise surface functionalization of magnetic nanoparticles to regulate their interfacial behavior is considered a key strategy to enhance their demulsification performance [18].

Surface engineering has been widely recognized as an effective approach to endow nanomaterials with new functionalities and improved performance. By introducing specific functional groups or polymer coatings, the dispersion stability, interfacial affinity, and overall performance of Fe_3_O_4_ nanoparticles can be significantly improved [19]. Among various strategies, constructing a core–shell structure with Fe_3_O_4_ as the magnetic core and a carbon layer as the intermediate shell (Fe_3_O_4_@C), followed by further surface modification, represents a promising approach [20]. The carbon layer not only protects the magnetic core from corrosion but also provides an ideal platform for subsequent functionalization. Polyethyleneimine (PEI), a cationic polymer rich in amine groups, can be grafted onto the surface of Fe_3_O_4_@C nanoparticles to fabricate PEI-functionalized magnetic carbon nanoparticles (P-MCN) [21]. This design integrates the advantages of magnetic recoverability, structural stability of carbon materials, and strong interfacial activity of PEI. The demulsification mechanism is attributed to the abundant positively charged amine groups on the PEI chains, which can strongly adsorb onto the negatively charged oil–water interfacial film via electrostatic interactions, thereby neutralizing droplet charges. Meanwhile, the strong interfacial affinity of P-MCN enables it to effectively replace bitumen at the interface, significantly weakening the interfacial film strength and stability [22,23,24], thus promoting droplet coalescence and phase separation.

**Figure 1:**
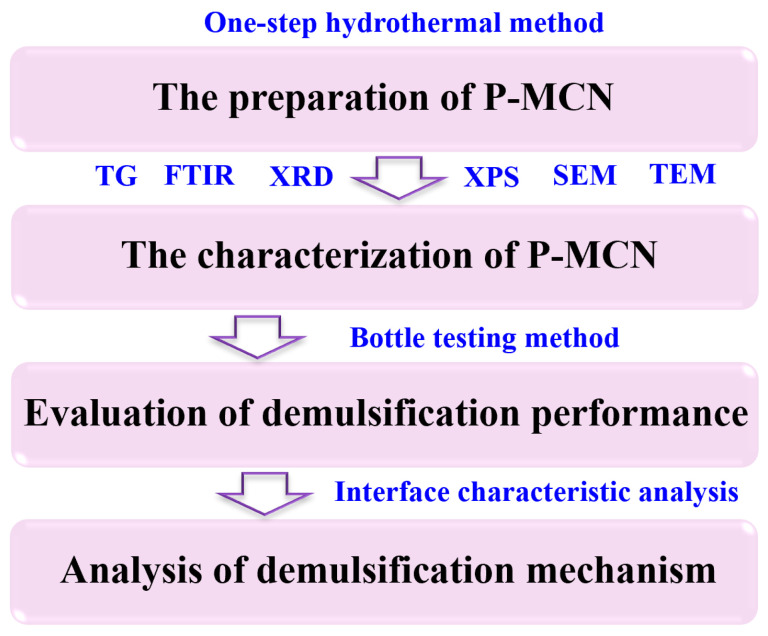
Research framework of this paper.

In summary, to address the challenge of highly stable heavy oil emulsions and develop an efficient and magnetically recoverable demulsifier, polyethyleneimine-functionalized Fe_3_O_4_@C nanoparticles (P-MCN) were successfully synthesized via a one-step hydrothermal method in this study. The chemical structure, crystalline properties, elemental composition, and morphology were systematically characterized by Fourier transform infrared spectroscopy (FT-IR), X-ray diffraction (XRD), X-ray photoelectron spectroscopy (XPS), scanning electron microscopy (SEM), and transmission electron microscopy (TEM). Subsequently, the demulsification performance of P-MCN under various conditions was systematically evaluated using the standard bottle test method [25]. Furthermore, the interfacial activity characterization was employed to elucidate the demulsification mechanism from both macroscopic and molecular perspectives (Figure 1). This study not only provides a high-performance material for heavy oil emulsion demulsification, but also offers important theoretical guidance and practical insights for the future design of highly efficient, environmentally adaptable, and industrially scalable magnetic nanodemulsifiers.

## 2. Experimental Section

### 2.1. Reagents and Materials

All chemicals were of analytical grade and used without further purification. Ethylene glycol and absolute ethanol were purchased from Tianjin Fuyu Fine Chemical Co., Ltd. (Tianjin, China). Sodium acetate anhydrous was obtained from Chengdu Jinshan Chemical Reagent Co., Ltd. (Chengdu, China). Toluene was supplied by Chongqing Chuandong Chemical Group Co., Ltd. (Chongqing, China). Iron(III) chloride hexahydrate and glucose were purchased from Shanghai Aladdin Biochemical Technology Co., Ltd. (Shanghai, China). Polyethyleneimine was obtained from Shanghai Macklin Biochemical Technology Co., Ltd. (Shanghai, China). Milli-Q water (resistivity of 18.2 MΩ·cm at 25 °C) was used throughout the experiments. Heavy oil samples were extracted from Indonesian oil sands via a solvent extraction method using toluene and heptane as solvents. The composition and content of heavy oil are shown in Table 1.

### 2.2. Preparation of P-Mcn

#### 2.2.1. Preparation of Carbon Nanoparticles

Briefly, 10 g of glucose was first dissolved in 100 mL of deionized water in a beaker under stirring to obtain a homogeneous solution. The mixture was then transferred into a Teflon-lined autoclave and sealed. The autoclave was placed in a muffle furnace and heated at 180 °C for 12 h. After cooling to room temperature, a brown-black suspension was obtained. The product was collected and washed five times with deionized water and absolute ethanol, respectively, followed by drying at 60 °C to yield brown-black carbon nanoparticles (abbreviated as CNS).

#### 2.2.2. Preparation of Magnetic P-Mcn Nanoparticles

Briefly, 2 g of FeCl_3_·6H_2_O was dissolved in 65 mL of ethylene glycol in a 100 mL beaker. Subsequently, 6 g of sodium acetate, 3 g of polyethyleneimine (PEI, 50 wt% aqueous solution), and 0.03 g of CNS were added, followed by vigorous stirring and ultrasonication for 30 min to ensure uniform dispersion. The homogeneous mixture was then transferred into a Teflon-lined stainless-steel autoclave and maintained at 200 °C for 6 h. After the reaction, the autoclave was naturally cooled to room temperature, and the obtained product was collected. The magnetic product was separated using an external magnet and washed with absolute ethanol 3–5 times. Finally, the product was dried in a vacuum oven at 60 °C for 12 h to obtain brownish-brown magnetic P-MCN nanoparticles (Figure S1).

### 2.3. Characterization of Structural Properties of P-Mcn

Fourier transform infrared spectroscopy (FT-IR, FTS 6000, Bio-Rad, San Francisco, CA, USA) was employed to identify the organic functional groups of carbon nanoparticles, magnetic carbon nanoparticles, and functionalized magnetic carbon nanoparticles. The spectra were recorded in the range of 4000–400 cm^−1^.

The crystalline structures of carbon nanoparticles, magnetic carbon nanoparticles, and functionalized magnetic carbon nanoparticles were characterized by X-ray diffraction (XRD, D8 Advance, Bruker, Mannheim, Germany) using Cu Kα radiation (λ = 0.15418 nm) as the X-ray source. The measurements were conducted over a 2θ range of 10–80° at a scanning rate of 5° min^−1^.

The surface elemental composition of P-MCN was analyzed by X-ray photoelectron spectroscopy (XPS, Escalab 250Xi, Thermo Fisher Scientific, Waltham, MA, USA) using monochromatic Al Kα radiation as the excitation source.

### 2.4. Evaluation of Demulsification Performance of P-Mcn

The demulsification performance of P-MCN in water-in-heavy oil (W/HO) emulsions was evaluated using the standard bottle test method in accordance with ASTM D2711-17 and SY/T 5281-2000, ensuring the reliability and reproducibility of the experimental data. Bitumen (1 g) was dissolved in 100 mL of toluene to prepare the oil phase, followed by emulsification with 10 mL of ultrapure water using an FJ 200-S homogenizer at 15,000 rpm for 5 min. The resulting W/HO emulsion (15 mL) was then allowed to stand for 12 h to reach equilibrium. Subsequently, 7.5 mL of the W/HO emulsion was transferred into a graduated cylinder, and P-MCN was added at a concentration of 500 ppm. A blank emulsion without P-MCN was used as a control. The demulsification performance of P-MCN was investigated and evaluated accordingly. The demulsification efficiency was assessed by measuring the volume of water separated from the W/HO emulsions. The dehydration efficiency (DE) was calculated according to Equation (1):

DE = *V_t_*/*V*_0_ × 100% (1)

where DE (%) is the dehydration efficiency, and *V_t_* and *V*_0_ represent the volume of separated water (mL) and the initial water (mL), respectively.

## 3. Results and Discussion

### 3.1. Characterization of Functionalized Magnetic Carbon Nanoparticles

**Figure 2:**
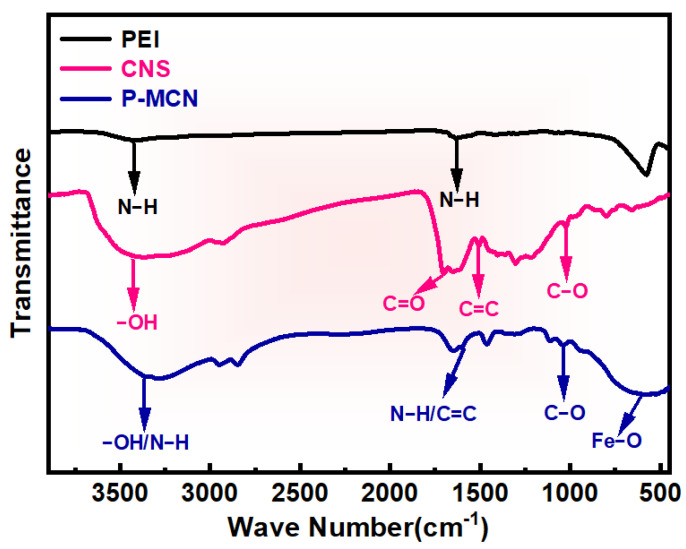
FTIR spectrum of PEI, CNS, P-MCN.

Figure 2 shows the FTIR spectra of CNS, PEI, and P-MCN. Significant differences in spectral features are observed among the three samples, indicating the reconstruction of surface functional groups during the functionalization process. The FTIR spectra of PEI, the broad band at 3300–3400 cm^−1^ corresponds to the stretching vibration of N-H groups, while the band located at approximately 1580–1650 cm^−1^ is attributed to the bending vibration of amino groups, which is a characteristic feature of polyethyleneimine [26]. In the spectrum of CNS, a broad absorption band centered at approximately 3430 cm^−1^ is observed, which is attributed to the stretching vibration of hydroxyl groups and other oxygen-containing functionalities generated during the hydrothermal carbonization of glucose. In addition, the absorption bands located around 1700 cm^−1^ and 1050–1200 cm^−1^ can be assigned to the stretching vibrations of carbonyl (C=O) and C-O groups, respectively, indicating the presence of abundant oxygen-containing functional groups on the CNS surface. And the absorption band near 1600 cm^−1^ is associated with the skeletal vibration of aromatic C=C structures formed during the carbonization process These functional groups provide active sites for subsequent surface modification and nanoparticle assembly [27]. The FTIR spectra of P-MCN, the FTIR spectrum of P-MCN exhibits several notable changes. The broad absorption band around 3430 cm^−1^ becomes stronger and broader compared with that of CNS, which can be attributed to the overlapping stretching vibrations of O-H groups from CNS and N-H groups introduced by PEI. Meanwhile, the enhanced absorption in the 1580–1620 cm^−1^ region is assigned to the combined contribution of N-H bending vibrations from PEI and aromatic C=C skeletal vibrations of the carbon framework, indicating the successful introduction of amino functionalities onto the particle surface. In addition, a distinct absorption peak appears at approximately 580 cm^−1^, corresponding to the Fe-O stretching vibration of Fe_3_O_4_ nanoparticles, which confirms the successful formation of the magnetic core [28,29]. These results collectively demonstrate that Fe_3_O_4_ and PEI have been successfully introduced onto the surface of magnetic carbon nanoparticles, forming stable hydrogen bonding and coordination structures. This provides a solid foundation for enhanced polarity and improved interfacial adsorption capability at the oil–water interface.

**Figure 3:**
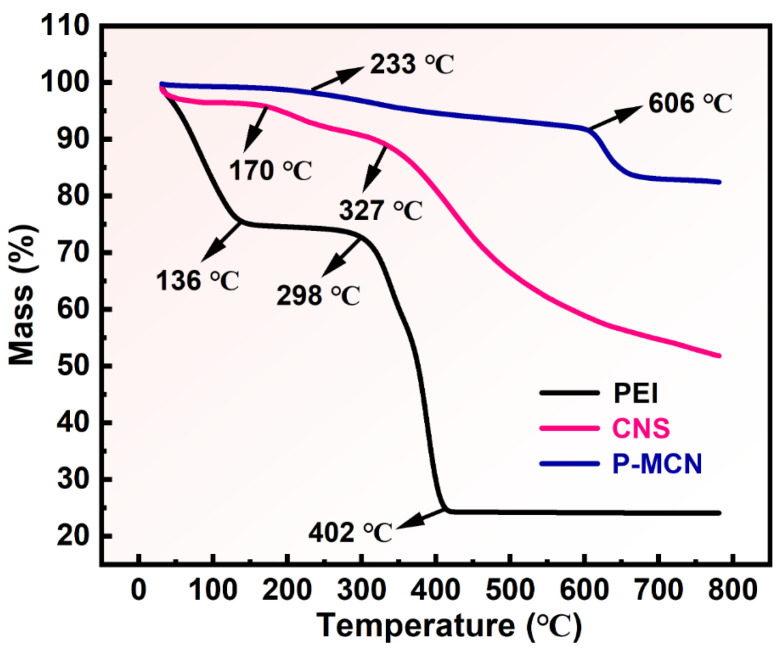
TGA spectrum of PEI, CNS, P-MCN.

In industrial applications, demulsifiers are required to maintain structural stability over a wide temperature range to withstand the high-temperature conditions encountered during crude oil extraction and processing. Therefore, it is necessary to systematically evaluate the thermal stability of the prepared materials. Figure 3 presents the thermogravimetric (TGA) curves of PEI, CNS, and P-MCN.

As shown in Figure 3, all three samples exhibit slight weight loss in the low-temperature region (room temperature to approximately 150 °C). For PEI, a noticeable weight loss occurs from room temperature to 136 °C, which is mainly attributed to the evaporation of physically adsorbed water and a small amount of low-molecular-weight species. In contrast, the TGA curves of CNS and P-MCN display relatively slow mass loss in this temperature range, indicating their superior structural stability. With further increasing temperature to around 300 °C, PEI undergoes a second-stage weight loss starting at approximately 298 °C, while CNS enters a pronounced thermal decomposition stage at about 327 °C. This behavior is mainly associated with the cleavage of functional groups such as C–H, C–O, and O–H within the organic framework. When the temperature exceeds 298 °C, the mass of PEI decreases rapidly and stabilizes after approximately 402 °C, leaving a residual mass of about 23%, indicating severe decomposition of its organic components at elevated temperatures. In comparison, the TGA curves of CNS exhibits a more gradual weight loss process and retains approximately 52% of its mass at 800 °C, demonstrating relatively good thermal stability of the carbon framework. Notably, the TGA curves of P-MCN exhibits the highest thermal stability among the three samples. Its main weight loss stage is delayed to around 606 °C, and more than 80% of its mass is retained at 800 °C. This superior performance can be attributed to the synergistic effect between the magnetic components and the carbon matrix, which significantly enhances the thermal resistance of the material. Overall, P-MCN demonstrates excellent thermal stability and maintains high structural integrity under high-temperature conditions, meeting the requirements for industrial demulsification processes and providing strong support for its application in high-temperature heavy oil emulsion systems.

**Figure 4:**
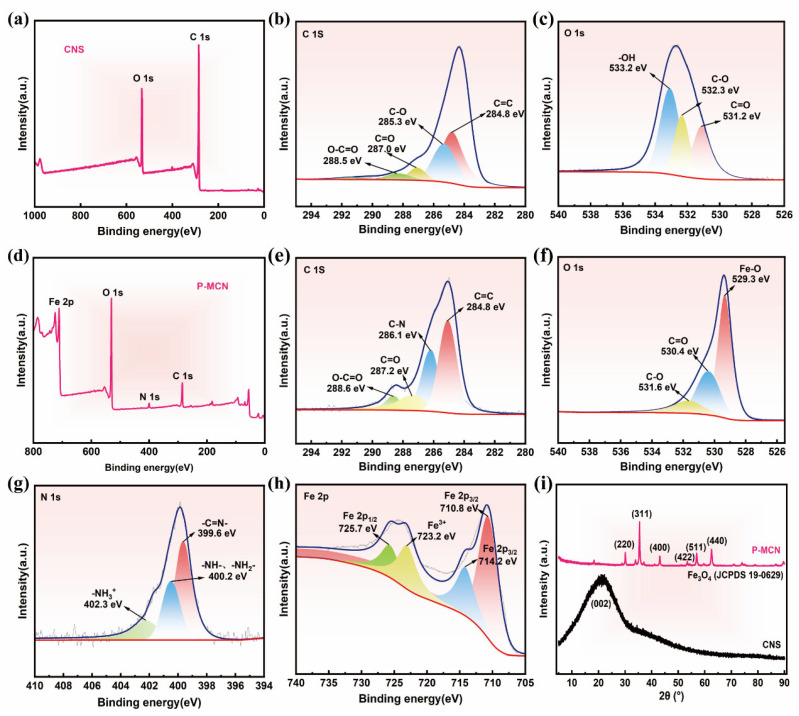
XPS spectrum of CNS and P-MCN: (**a**) survey scan of CNS showing the presence of C1s, O1s, (**b**) high-resolution C1s spectrum of CNS, (**c**) O1S spectrum of CNS, (**d**) survey scan of P-MCN revealing C1s, N1s, O1s, Fe2p, (**e**) C1s spectrum of P-MCN, (**f**) O1s spectrum of P-MCN, (**g**) N1s spectrum of P-MCN, (**h**) Fe2p spectrum of P-MCN, (**i**) XRD spectrum of CNS and P-MCN.

To further confirm PEI functionalization and the evolution of the interfacial chemical environment, X-ray photoelectron spectroscopy (XPS) was employed to analyze the elemental composition and chemical states of the samples. As shown in Figure 4, the survey spectra reveal that CNS mainly contains C and O elements, whereas P-MCN exhibits distinct Fe 2p, O 1s, and N 1s signals, confirming the successful incorporation of Fe_3_O_4_ and PEI.

In the high-resolution Fe 2p spectrum, the characteristic peaks at ~710.5 eV (Fe 2p_3_/_2_) and ~724.0 eV (Fe 2p_1_/_2_), along with their satellite peaks, are consistent with the mixed valence states of Fe^2+^/Fe^3+^ in Fe_3_O_4_. Compared with pure Fe_3_O_4_, a slight positive shift in the Fe 2p peaks is observed in P-MCN, indicating electronic interactions between the amine groups of PEI and the Fe_3_O_4_ surface, which alter the local electron density [18,30,31]. In the C 1s spectrum (Figure 4b), CNS exhibits well-defined C–C/C=C (284.6 eV) and C–O (286.3 eV) peaks. In contrast, additional components corresponding to –C–N/–N–C and N–C=O are observed in P-MCN (Figure 4e), suggesting the involvement of nitrogen species from PEI in surface chemical bonding. The N 1s spectrum (Figure 4g) further displays two characteristic peaks at ~399.6 eV (–NH_2_) and ~401 eV (–NH–), which are typical fingerprints of PEI [32,33,34]. The O 1s spectrum shows multiple components corresponding to Fe–O, C–O, and C=O species(Figure 4f), indicating the coexistence of various oxygen-containing functionalities and reflecting complex interfacial coordination and electronic interactions [35]. These XPS results collectively demonstrate that PEI is successfully grafted onto the surface of both the carbon matrix and Fe_3_O_4_, leading to significant reconstruction of surface chemical bonds. This modification enhances the surface polarity and is expected to improve competitive adsorption at the oil–water interface during demulsification. Figure 4i presents the XRD patterns of CNS and P-MCN. The diffraction peak of CNS at ~26° corresponds to the (002) plane of graphitic carbon, indicating a relatively high degree of graphitization [36]. For P-MCN, the diffraction pattern contains both the broad amorphous carbon peak and all characteristic peaks of Fe_3_O_4_. Notably, the Fe_3_O_4_ peaks show no obvious shift, with only a slight decrease in intensity. This suggests that the PEI coating reduces surface scattering intensity, while the crystal structure of Fe_3_O_4_ remains well-preserved during the functionalization process without phase transformation or lattice distortion. In addition, no impurity peaks are observed, indicating that no extra crystalline phases were introduced during synthesis. These results confirm that the magnetic core maintains excellent crystallinity and structural stability, which is essential for achieving rapid magnetic response and stable recyclability.

**Figure 5:**
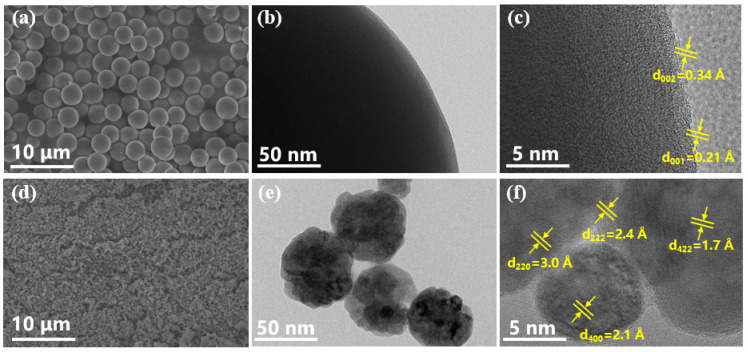
(**a**) SEM images of CNS and (**d**) P-MCN, (**b**) TEM images of CNS and (**e**) P-MCN, (**c**) HR-TEM images of CNS and (**f**) HR-TEM images of P-MCN.

Figure 5 presents the morphology and microstructural characteristics of CNS and the modified P-MCN. SEM images (Figure 5a,d) show that CNS exhibits a relatively uniform spherical structure with a narrow particle size distribution and a smooth surface. In contrast, P-MCN (Figure 5d) exhibits a noticeably rougher surface, accompanied by a certain degree of aggregation. This can be attributed to the introduction of inorganic nanoparticles and surface polar groups, which enhance the interparticle interactions. The TEM images (Figure 5b,e) further elucidate the internal structure of the samples. The TEM image of CNS (Figure 5b) displays a uniform and compact structure with clear boundaries. In contrast, the TEM image of P-MCN (Figure 5e) exhibits noticeable particle aggregation and interparticle adhesion, accompanied by blurred particle edges, suggesting the formation of a surface coating layer. The HR-TEM images (Figure 5c,f) provide more direct insights into the crystalline structure. In the HR-TEM image of CNS (Figure 5c), locally ordered lattice fringes can be observed, with interplanar spacings of approximately 0.34 nm and 0.21 nm, corresponding to the (002) and (100) planes of graphitic carbon [37], respectively. This indicates that the material is predominantly amorphous carbon with a certain degree of graphitization. In contrast, the HR-TEM image of P-MCN (Figure 5f) exhibits well-defined lattice fringes with interplanar spacings of approximately 0.17 nm, 0.21 nm, 0.24 nm, and 0.30 nm, corresponding to the (422), (400), (222), and (220) planes of Fe_3_O_4_ [38], respectively. This clearly confirms that magnetic Fe_3_O_4_ nanoparticles are successfully anchored onto the carbon matrix. Furthermore, a distinct shell layer is observed at the particle edges of the modified sample, indicating the formation of an organic coating on the Fe_3_O_4_ surface, thereby constructing a core–shell structure. Such a configuration enhances interfacial activity and is expected to promote adsorption and diffusion at the oil–water interface.

### 3.2. Demulsification Performance of P-Mcn

**Figure 6:**
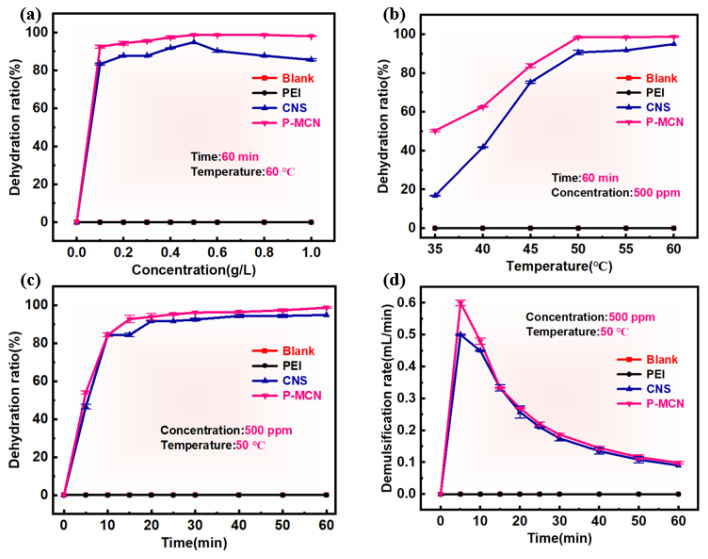

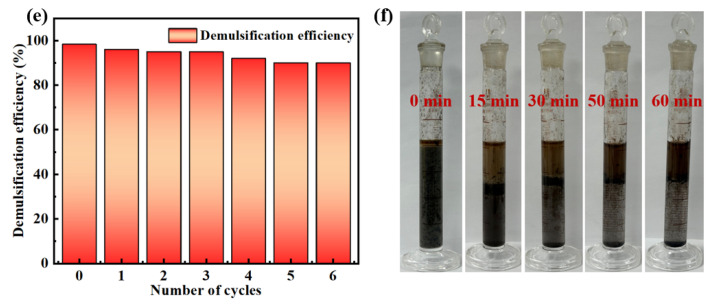
Effect of different operating conditions: (**a**) demulsifier concentration, (**b**) demulsification temperature, (**c**) settling time on dehydration performance of demulsifiers in W/HO emulsions, (**d**) dehydration rate of demulsifiers, (**e**) dehydration ratio IAA-stabilized W/HO by P-MCN (500 ppm) with different cycle times and (**f**) dehydration photographs.

The demulsification performance of P-MCN in W/HO emulsions was evaluated using the bottle test method. The effects of demulsifier dosage, temperature, and settling time on the demulsification performance were systematically investigated, as shown in Figure 6. Figure 6a–d present the dehydration performance of different demulsifiers (Blank, PEI, CNS, and P-MCN) for W/HO emulsions under various conditions. As shown in Figure 6a, the dehydration efficiency increases significantly with increasing demulsifier dosage. When the concentration exceeds 400 ppm, P-MCN achieves nearly 100% dehydration efficiency, which is significantly higher than that of unmodified CNS and PEI. In contrast, PEI alone and the blank sample exhibit negligible dehydration performance, indicating that electrostatic adsorption of polymer chains alone is insufficient to achieve efficient demulsification of water-in-heavy oil emulsions. In comparison, the magnetic carbon nanocomposite structure demonstrates significant advantages in interfacial adsorption and polar interactions. Moreover, when the demulsifier dosage reaches 500 ppm, the demulsification performance of P-MCN tends to level off, whereas the demulsification efficiency of CNS exhibits a declining trend. This indicates that, when the demulsifier concentration exceeds its critical micelle concentration (CMC), the demulsification efficiency decreases. This is attributed to the formation of micelles in the aqueous phase, which reduces the interfacial activity of the demulsifier, thereby leading to poorer dehydration performance. These results demonstrate that the optimal demulsification performance of CNS and P-MCN is achieved at their critical micelle concentration (CMC), with an optimal dosage of 500 ppm.

Figure 6b illustrates the effect of temperature on the demulsification performance. At a fixed dosage of 500 ppm and a settling time of 60 min, increasing the temperature from 35 °C to 60 °C does not lead to any noticeable change in the demulsification performance of the blank sample and PEI-treated emulsion, indicating that the W/HO emulsions remains stable without effective demulsification. As the temperature increases from 35 to 50 °C at a settling time of 60 min, the dehydration efficiency of P-MCN in W/HO emulsions increases from 50% to 98.33%, whereas that of CNS only rises from 16.7% to 90%. The results demonstrate that CNS exhibits lower dehydration efficiency than P-MCN, while P-MCN achieves nearly complete dehydration, confirming its superior demulsification performance. This can be attributed to the elevated temperature, which promotes the disruption of the IAA interfacial film and enhances molecular mobility, thereby accelerating oil–water separation. Meanwhile, the presence of amine and hydroxyl groups on the surface of P-MCN strengthens interactions with polar groups on the droplet surface, thereby further destabilizing the oil–water interfacial film.

Figure 6c shows the variation in dehydration efficiency with time. At a fixed dosage of 500 ppm and a temperature of 50 °C, P-MCN exhibits a rapid demulsification trend within the first 10 min, achieving a dehydration efficiency of 80%. The efficiency then gradually increases and stabilizes after 30 min, ultimately reaching 98.33% at 60 min, indicating nearly complete demulsification. In contrast, the unmodified CNS exhibits a slightly lower demulsification rate, indicating that PEI functionalization significantly enhances the interfacial demulsification performance and dispersion stability of the particles. Figure 6d compares the demulsification rates of different demulsifiers. P-MCN exhibits the highest demulsification rate in the initial stage, reaching a peak value of approximately 0.6 mL min^−1^ within the first 10 min, followed by a gradual decline, indicating that the demulsification process predominantly occurs at the early stage. Additionally, a comparison of P-MCN’s demulsification efficiency with other reported magnetic demulsifiers (Table 2) revealed that P-MCN is competitive and often superior.

Figure 6e shows the variation in dehydration ratio IAA-stabilized W/HO by P-MCN (500 ppm) with different cycle times. The demulsification efficiency remained above 92% after six consecutive cycles, indicating that the active interfacial adsorption sites of P-MCN were largely preserved during reuse. This observation indirectly suggests that severe degradation or detachment of the surface functional layer did not occur under the experimental conditions. Although the demulsification efficiency remained relatively stable during the recycling experiments, the structural evolution of P-MCN after repeated use was not systematically investigated in the present work. Therefore, the integrity of the PEI coating and the potential leaching of Fe or nitrogen-containing species cannot be directly confirmed. Future studies will focus on post-recycling FT-IR, XPS and TEM characterization, together with elemental analysis of the treated aqueous phase, to evaluate the long-term structural stability and environmental safety of P-MCN.

Overall, P-MCN demonstrates superior demulsification efficiency and rate in W/HO emulsions. This outstanding performance can be attributed to the synergistic effects of enhanced interfacial hydrophilicity induced by surface amination, strong interfacial adsorption arising from the high specific surface area of nanoparticles, and the magnetic-assisted droplet aggregation and separation. These results highlight the great potential of P-MCN as an efficient and environmentally friendly demulsifier for rapid separation in complex heavy oil–water systems.

### 3.3. The Demulsification Mechanism

**Figure 7:**
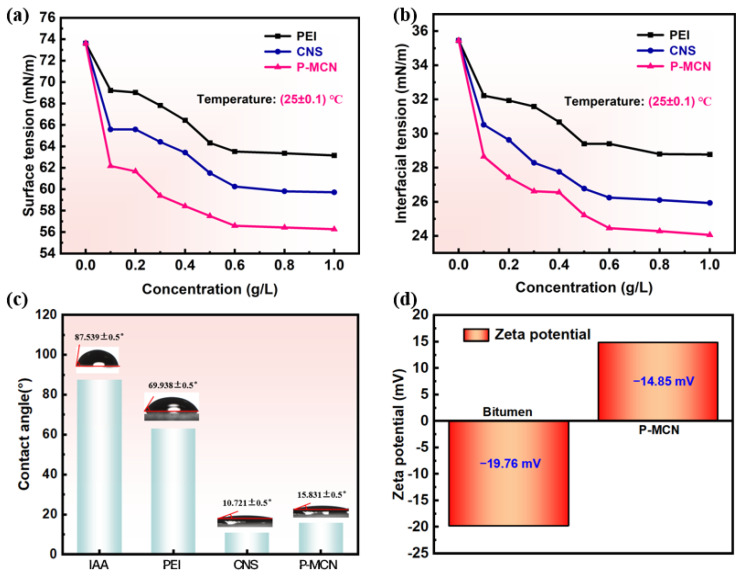
(**a**) Surface tension of demulsifier aqueous solution, (**b**) interfacial tension of the oil–water with PEI, CNS, P-MCN and bitumen, (**c**) changes in three-phase contact angle, (**d**) zeta potential values of bitumen and P-MCN.

To elucidate the demulsification mechanism of P-MCN in W/HO emulsions, it is essential to first consider the role of bitumen in emulsion stabilization. Previous studies have demonstrated that bitumen acts as the primary stabilizing component in W/HO emulsions due to its amphiphilic nature and strong interfacial activity. Bitumen molecules can adsorb at the oil–water interface and form a rigid and viscoelastic interfacial film through π–π stacking, hydrogen bonding, and van der Waals interactions, thereby preventing droplet coalescence and maintaining emulsion stability [39]. Based on this understanding, the demulsification behavior of P-MCN was systematically analyzed through interfacial property measurements. As shown in Figure 7a,b, both surface tension and oil–water interfacial tension decrease with increasing concentration at 25 °C. Notably, P-MCN exhibits the most pronounced reduction, indicating its superior interfacial activity. The significant decrease in interfacial tension effectively lowers the energy barrier between droplets, thereby facilitating droplet deformation and coalescence, which accelerates emulsion destabilization. Furthermore, contact angle measurements (Figure 7c) reveal that P-MCN exhibits the lowest contact angle among all samples, demonstrating its enhanced hydrophilicity. This property enables P-MCN to rapidly migrate and adsorb at the oil–water interface, leading to more efficient interfacial coverage and competitive displacement of bitumen molecules. In addition to interfacial activity, electrostatic interactions also play a crucial role in the demulsification process. As shown in Figure 7d, bitumen carries a negative charge (−19.76 mV), whereas P-MCN is positively charged (+14.85 mV), resulting in strong electrostatic attraction. This interaction promotes the directional adsorption of P-MCN onto the negatively charged interfacial film and disrupts the bitumen-stabilized network structure. The combined effects of electrostatic attraction and hydrogen bonding further weaken intermolecular interactions within the interfacial film, leading to its collapse.

**Figure 8:**
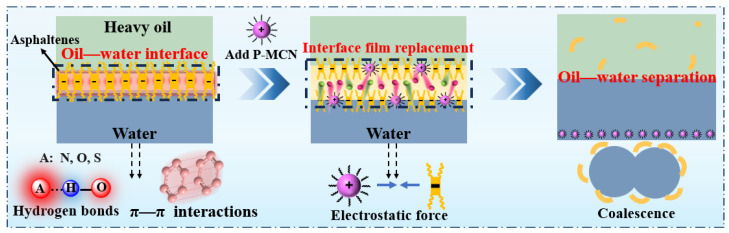
Schematic diagram of demulsification mechanism.

To sum up, as shown in Figure 8, the demulsification mechanism of P-MCN can be attributed to a synergistic effect of enhanced interfacial activity and strong electrostatic interactions. On the one hand, P-MCN reduces the interfacial free energy and promotes droplet coalescence; on the other hand, it disrupts the rigid bitumen interfacial film, transforming it from a compact and stable structure into a loose and fragile one, and then promote the coalescence of emulsion drops, thereby significantly improving the demulsification efficiency of W/HO emulsions.

## 4. Conclusions

In this study, polyethyleneimine-functionalized magnetic carbon nanoparticles (P-MCNs) were successfully constructed via a surface functionalization strategy for the efficient demulsification of water-in-heavy oil (W/HO) emulsions. The experimental results demonstrate that, at a demulsifier dosage of 500 ppm and a temperature of 50 °C, P-MCN achieves a high dehydration efficiency of up to 98.33% within 60 min, significantly outperforming unmodified magnetic carbon nanoparticles (CNSs) and pure polyethyleneimine (PEI). Mechanistic investigations reveal that the superior demulsification performance of P-MCN is primarily attributed to its high interfacial activity and unique structural synergy. On the one hand, the abundant amine groups of PEI and the carbon shell structure endow the material with excellent hydrophilicity and interfacial adsorption capability, enabling rapid migration to the oil–water interface. On the other hand, P-MCN effectively disrupts the stable interfacial film formed by bitumen through noncovalent interactions such as electrostatic attraction and hydrogen bonding, thereby promoting droplet aggregation and coalescence and ultimately achieving efficient oil–water separation. In addition, P-MCN exhibits excellent thermal stability and magnetic separability, providing important support for its practical application in complex heavy oil–water systems. This study not only provides a novel functionalized magnetic nanomaterial for efficient demulsification of W/HO emulsions, but also offers valuable theoretical insights and engineering guidance for the future design of highly efficient, low-temperature-responsive, and recyclable demulsifiers.

## Figures and Tables

**Table 1:** The composition and content of heavy oil.

Sample	Viscosity	Density	Asphaltene Content	Resin Content	Water Content
heavy oil	24,535 mPa·s	1038.7 kg·m^−3^	27.1 wt%	26.62 wt%	2 wt%

**Table 2:** Comparison of demulsification performance of nano demulsification materials.

Demulsifier	Emulsions	Dosage (ppm)	Temperature (°C)	Time (min)	Demulsification Efficiency (%)	Reusability	Ref
P-MCN	water/heavy oil	500	50	60	98.33	6/92	This work
P[IL]@MCs	water/crude oil	1000	70	120	99.9	4/89	[39]
Fe_3_N@F	water/crude oil	150	45	150	89.4	4/70	[40]
M@NH_2_ MNPs	water/crude oil	250	25	210	95	9/82	[29]
PQA-PDA@Fe_3_O_4_	water/crude oil	200	25	30	90.5	5/85.6	[18]
Fe_3_O_4_@C-F	water/crude oil	800	65	90	91.68	6/60	[41]
CF	water/crude oil	2500	25	60	90	4/65	[42]
MND	water/crude oil	1900	80	5	100	4/94.64	[43]

## Data Availability

The original contributions presented in this study are included in the article/Supplementary Materials. Further inquiries can be directed to the corresponding author.

## Supplementary Materials

The following supporting information can be downloaded at: https://www.mdpi.com/article/10.3390/ma19122584/s1, Figure S1: Physical photos of different demulsification materials.
