# Supplementary material for: Functionalized Magnetic Carbon Nanoparticles Efficiently Break Water-in-Heavy Oil Emulsions
Source: Materials (Basel). 2026 Jun 15;19(12):2584. doi: 10.3390/ma19122584 (PMC13303635; doi:10.3390/ma19122584)
Supplement: Supplementary file 1 [file materials-19-02584-s001.zip › materials-4332811-supplementary.pdf]

Supporting Information

Functionalized Magnetic Carbon Nanoparticles Efficiently Break Water-in-Heavy Oil Emulsions

Jinlong Gao [1,†], Lulu Yan [2,†] and Jun Ma [2,*]

[1] College of Energy and Chemical Engineering, Shaanxi Polytechnic University, Xianyang 712000, China
[2] Department of Chemical Engineering, School of Chemistry and Chemical Engineering, Guizhou University, Guiyang 550025, China
* Correspondence: jma3@gzu.edu.cn
† These authors contributed equally to this work.

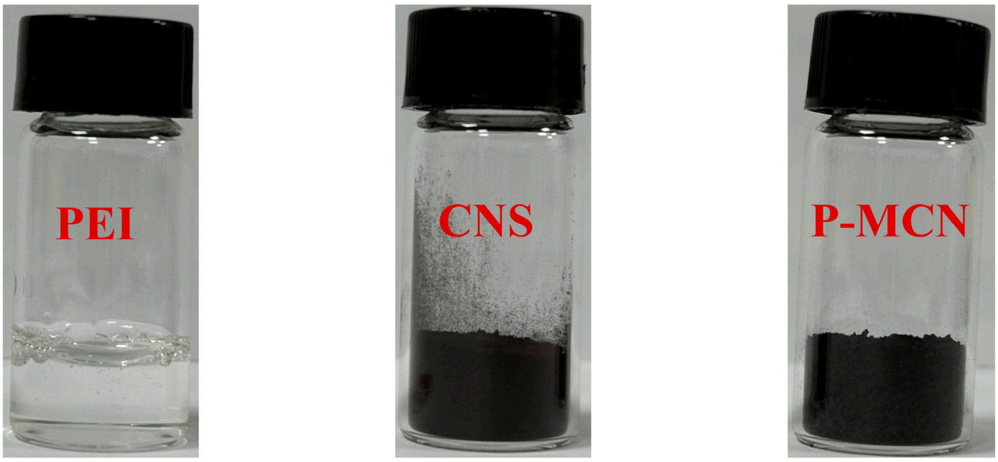

Figure S1. Physical photos of different demulsification materials.
